# Encapsulation of Allyl Isothiocyanate by Freeze- and Spray-Drying: Effects on Retention and Sensory Perception in Sodium-Reduced Soups

**DOI:** 10.3390/foods14223810

**Published:** 2025-11-07

**Authors:** Emily Dolan, Nicoletta Faraone, Matthew B. McSweeney

**Affiliations:** 1 School of Nutrition and Dietetics, Acadia University, Wolfville, NS B4P 2R6, Canada; 161731d@acadiau.ca; 2 Chemistry Department, Acadia University, Wolfville, NS B4P 2R6, Canada; nicoletta.faraone@acadiau.ca

**Keywords:** maltodextrin, gum Arabic, trigeminal sensations, consumer perception, horseradish, saltiness

## Abstract

Allyl isothiocyanate (AITC) has been shown to enhance perceived saltiness in food products; however, it is also associated with a pungent and spicy flavour. The objective of this study was to assess the encapsulation of AITC with maltodextrin (MD) and gum Arabic (GA) using spray-drying (SD) and freeze-drying (FD) techniques, with and without the addition of a surfactant. Furthermore, the different encapsulated formulations were evaluated for their impact on sensory properties when added to soups. In total, twelve different treatments were investigated. The physicochemical characteristics (i.e., encapsulation efficiency, surface oil content, capsule morphology, and moisture content) and sensory properties (i.e., hedonic scales and rate-all-that-apply) of the encapsulated AITC particles were analyzed. Gas chromatography revealed low AITC retention in all FD formulations, while SD formulations with surfactants achieved up to 136.71 mg AITC/g powder. Sensory trials were conducted on eight formulations added to tomato soup (0.500 mg AITC/100 mL) (SD trial: *n* = 79, and FD trial: *n* = 93). FD resulted in relatively low AITC retention (with and without surfactants), while SD with surfactants led to higher AITC retention. None of the formulations significantly impacted the saltiness perception of the soups. FD soups significantly enhanced thickness, creaminess, and tomato flavour, increasing overall liking. This is the first study to evaluate the sensory properties and cross-modal interactions of encapsulated AITC. Further studies are needed to continue exploring the sensory properties, its release behaviour, overall stability, and shelf life of encapsulated AITC.

## 1. Introduction

Recent research has placed greater emphasis on non-communicable diseases (NCDs) to enhance the overall health and well-being of the human population. Cardiovascular disease (CVD), cancers, chronic respiratory diseases, and diabetes have been of interest to public health authorities as these conditions together contribute to “80% of all premature deaths” attributed to NCDs and represent a substantial proportion of total deaths [1]. The ‘25 *by* 25’ target was adopted in 2012 by the United Nations (UN) at the World Health Assembly in 2012, aiming to reduce “premature mortality from [NCDs]” by 25% by the year 2025 [2].

A diet high in sodium is a risk factor for CVD [3,4,5]. In 2017, an estimated 17.8 million deaths were attributed to CVD globally, with 2.7 million specifically being associated with high dietary salt intake [6]. Reducing sodium intake has been identified as a method to decrease CVD risk, and it may help those individuals at higher risk; those who are older, overweight, obese or have elevated blood pressure [7]. Food reformulation has been identified as one strategy to reduce sodium intake at a population level. It is estimated that more than 75% of individuals’ sodium intake comes from food prepared outside the home [8]. While only about 180 to 230 mg of sodium per day is physiologically necessary, removing excess salt from food formulation is challenging as it is essential to many products’ flavour, texture, and stability [2]. Salt or sodium chloride is an important ingredient in the food industry as it enhances flavour and is essential in food preservation, which can challenge food reformulation [9]. High levels of sodium can be found in many food products including soups, breads, deli meats, and frozen meals, which are consumed regularly by many consumers. Specifically, past studies have identified that removing salt and sodium from products can decrease flavour complexity [10] and reduce consumer acceptance [11].

Recently, new techniques have been explored to reduce salt content and, in turn, sodium content in different foods [12]. Alternative salts, including potassium chloride, have been implemented as alternatives to create a salty taste in food products; however, they also lead to metallic, bitter, and sour flavours [13], which are disliked by consumers [14], and they have been linked stomach bloating and hyperkalemia. Other strategies used to reduce the amount of sodium in food products include stealth reduction, modification of salt crystals, saltiness potentiation, and government initiatives promoting the consumption of less sodium [15]. Another approach is based on multisensory integration. Although the underlying mechanisms are not fully understood, studies have demonstrated that certain aromas and compounds can enhance the perceived saltiness in products through cross-modal interactions [9]. The fifth cranial nerve (i.e., trigeminal nerve) innervates various regions of the face and mouth, including the oral cavity, and carries important sensory information related to “touch, temperature, and pain” [16,17]. While it primarily serves as a protective mechanism to alert against the consumption of irritants and poisonous substances, it also plays an essential role in flavour perception [17]. Flavour is a combination of taste (five basic tastes), aroma and trigeminal sensations [18]. Trigeminal compounds, such as spices, can evoke sensations that contribute to a product’s overall flavour perception, such as cooling, warmth, pungency, and tingling, in addition to taste and smell [17]. This sensation is called chemesthesis [19], and these trigeminal compounds or chemical irritants can impact overall flavour perception [20]. Recently, there has been a growing understanding of the role of transient receptor potential (TRP) channels in chemesthesis. Thermosensitive TRP channels, such as TRP vanilloid 1 (TRPV1) or TRP fixed hormone isoform (TRPA1), are activated by chemical irritants [21]. Due to their proximity, the TRPV1 and TRPA1 channels found in chemosensory nerves can indirectly influence taste perception [21,22], particularly concerning saltiness perception [23,24]. These chemical irritants include capsaicin (found in chili peppers) and allyl isothiocyanate (AITC) (found in horseradish, mustard, and wasabi) [17,21,24,25,26].

AITC is found in mustard and horseradish and possesses a sharp aroma and biting sensations [27]. This sensation is attributed to glucosinolate sinigrin (2-propenyl-glucosinolate) [27]. Past studies have identified that the trigeminal sensation experienced due to AITC depends on the concentration and the food matrix (differed based on oil vs. water carriers) [28]. Both AITC and capsaicin (another chemical irritant) have enhanced the saltiness intensity in sodium-reduced products. However, undesirable sensory characteristics like sourness, bitterness, metallic, and burning sensations often overshadow this effect, decreasing palatability [24,25]. AITC was explored in this study as it has been found to increase saltiness perception in past studies with Western consumers [25]. Furthermore, AITC and its cross-modal have not been thoroughly investigated in comparison to capsaicin. The volatility, reactivity, low solubility, and powerful odours of many spicy compounds limit their use in the food industry [29]. AITC is an oil that is sparingly soluble in water, as it is an electrophile susceptible to attacks from nucleophiles such as water and amino groups [30]. These traits limit its application in complex food systems. Therefore, encapsulation may offer a solution to this problem as it is often used to preserve bioactive compounds by coating them in wall material, such as maltodextrin (MD), modified starch, or gum Arabic (GA) to enhance its stability and retain the active component [31]. Encapsulation offers protection from harsh external environmental conditions, enhances handling ability, and improves water solubility, making it practical for various uses in the food industry [32]. Two widely used techniques for encapsulation include spray-drying (SD) and freeze-drying (FD). Spray dryers work rapidly and at high temperatures, with inlet air temperatures of 150–250 °C and outlet air temperatures of 50–80 °C. Meanwhile freeze dryers work via sublimation; however, they have long processing times. The resulting product from both spray-drying and freeze-drying is based on the preparation methods and the wall materials used. Furthermore, surfactants can be used to help stabilize the emulsions. A past study used gum acacia to encapsulate AITC and then used it as an antimicrobial [33]. Furthermore, past studies have also utilized surfactants during the encapsulation of AITC [29,34]. This work will build on these studies by evaluating the use of Tween-20 and Tween-80 during encapsulation, using both objective measurements and sensory methodologies.

This study aims to build on past research to encapsulate AITC and evaluate its cross-modal interactions. As such, the objectives of this study were to encapsulate AITC using GA or MD as wall material, both with and without the presence of food-grade surfactants (i.e., Tween-20 (T20) or Tween-80 (T80)). Gum Arabic and maltodextrin were selected as wall materials because of their excellent film-forming ability, high solubility in water, emulsification properties, and ability to protect active compounds, and because they are economical and food-grade, making them practical for large-scale use. We selected freeze-drying (FD) and spray-drying (SD) as encapsulation techniques to evaluate the impact of different temperature conditions on AITC encapsulation. The chemical composition of the encapsulated AITC formulations was investigated. The selected products were then added to a food product (tomato soup) to evaluate its influence on acceptability and the food product’s sensory properties. Tomato soup was chosen for this study as it has been identified to have a high amount of sodium [35].

## 2. Materials and Methods

### 2.1. Encapsulation of the AITC

#### 2.1.1. Materials

AITC (food grade, liquid, CAS No. 57-06-7), phenyl isothiocyanate (PITC, liquid, CAS No. 103-72-0), Tween-20 (liquid, CAS No. 9005-64-5), and Tween-80 (food grade, liquid, CAS No. 9005-65-6) were purchased from Sigma-Aldrich (Oakville, ON, Canada), gum Arabic (GA) powder (TexturestarAnhui, China; food grade, powder) and maltodextrin (MD) (LD Carlson, Kent, OH, USA; food grade, powder).

#### 2.1.2. Allyl Isothiocyanate Emulsion and Microcapsules

Initially, 10 g of wall material (MD or GA) was added to 490 mL of deionized water and then stirred overnight at room temperature. After approximately 24 h, 10 mL of AITC was added to the solution and, if applicable, 500 µL of the surfactants (T20 or T80) were added. The emulsion was immersed in an ice bath and homogenized for 30 m at 60% amplitude intensity using Fisherbrand™ Model 505 Sonic Dismembrator (Fisher Scientific, Pittsburgh, PA, USA).

Half of the emulsion was separated and was stored in 15 mL Falcon tubes at –80 °C in preparation for subsequent freeze-drying. After about 48 h at –80 °C, the product was freeze-dried using the VirTis Benchtops Pro freeze dryer equipped with the Omnitronics™—9 L Benchtop Freeze Dryer system and 12-Port Acrylic Vertical Drum Manifold (SP Scientific, Stone Ridge, NY, USA). Falcon tubes were covered by Kimwipes^®^ and carefully placed in Wide Mouth Filter Seal Flasks. The freeze dryer was set to Auto settings, maintaining an approximate pressure of 200 mT and a system temperature of –46 °C. Once the freeze-drying process was completed, the freeze-dried products were stored in a double Ziploc bag at −20 °C for later analysis.

The other half of the emulsion portions were spray-dried (Labfreez Instruments Group Co., Ltd., Beijing, China) according to the following parameters: 100% air intake, 130 °C inlet temperature, 90–80 °C outlet temperature, 15% feed rate, and approximate 4.5 mL/min flow rate. The spray-dried product was stored in a double Ziploc bag at −20 °C for later analysis.

#### 2.1.3. Percent Surface Oil Content

The percent surface oil content of the encapsulated products was evaluated in triplicate according to Bae and Lee [36] with modifications. Approximately 0.5 g of the encapsulated powders was weighed using an analytical balance and was added to 4 mL of hexane in 30 mL glass vials. Vials were then vortexed for two m and vacuum-filtered. Microcapsules were rinsed just once with 4 mL of hexane and dried at 60 °C for approximately 2 h. The samples were then weighed, and the percentage change between the initial and final weights was calculated using [(final weight − initial weight)/(initial weight)] × 100.

#### 2.1.4. AITC Quantification

The amount of AITC retained within the matrix was determined in triplicate, as described by Ko et al. [29] with modifications. Approximately 1 g of the spray-dried encapsulated powders and 0.5 g of the freeze-dried powders were each dispersed in 4 mL of deionized water in Falcon tubes. Then, 4 mL of hexane was added, and the mixture was mixed using a vortex mixer for 1 min. The Falcon tubes containing the mixtures were placed in a sonicator for 30 m at approximately 30 °C. The extracted mixtures were then centrifuged at 3000 rpm for 10 min, and the resulting supernatants were removed and stored in sealed glass vials at −20 °C.

The amount of AITC was quantified using gas chromatography with a flame-ionization detector (GC-FID). AITC standard solutions were prepared in hexane at different concentrations (31.25–3000 ng/µL) to build a calibration curve (Supplementary Materials Figure S1). PITC was chosen as the internal standard [29] at 160 ng/µL. 1 µL of each solution containing the PITC internal standard and standard AITC solution samples was injected in an Agilent 7890B GC System and Agilent 59,778 GC/MSD/FID (Agilent Technologies, Santa Clara, CA, USA) equipped with a Flame Ionization Detector using a PAL Autosampler (PAL RSI 85, PAL Auto Sampler System, Santa Clara, CA, USA) and an HP-5 ms Ultra Inert fused silica capillary column (30 m × 250 μm × 0.25 μm) (Agilent Technology Ltd., Santa Clara, CA, USA). The split ratio was set at 1:10, and the carrier gas was helium at a 1 mL/min flow rate. The temperature for the oven was set at 50 °C and held for 5 m. Then, the temperature was increased to 70 °C at a rate of 5 °C/min, followed by an increase to 230 °C at 20 °C/min.

After generating the calibration curve, 1 g of the powder was added to a Falcon tube containing 4 mL of deionized water. Then, 4 mL of hexane was added, and the mixture was vortexed for 1 m. The solution was then sonicated for 30 m in an ultrasonic bath at 30 °C. The mixture was centrifuged at 3000 rpm for 10 m, and the supernatant layer was collected. Samples were spiked with the internal standard and injected into the GC-FID system according to the conditions described above. The total concentration of AITC in the encapsulated sample was determined using the equation generated from the calibration curve.

#### 2.1.5. Scanning Electron Microscopy

The encapsulated powder formulations were analyzed using a scanning electron microscope. The samples were secured to the platforms using double-sided adhesive tape covered with each powder. Samples were coated using a Polaron SC7640 sputter coater using automatic settings (Quorom Technologies, Lewes, UK). The coating was a mixture of gold and palladium at a [6:4] ratio. Photographs were obtained using a JEOL LV-5900 SEM (JEOL U.S.A., Peabody, MA, USA) at the Acadia Centre for Microstructural Analysis (ACMA, Acadia University, Wolfville, NS, Canada).

#### 2.1.6. Moisture Content of AITC Microcapsules

Approximately 0.5 g of each powder was weighed using an analytical balance and placed in a round metal dish. Dishes were then kept at 105 °C for 18 h. After drying, each sample was reweighed, and the moisture content was calculated as the percentage between the initial and dry weights calculated using [(dried weight − initial weight)/(initial weight)] × 100. The process was completed in triplicate.

### 2.2. Sensory Evaluation

Two sensory trials were conducted. One evaluated four soups with different freeze-dried AITC formulations (GA-FD, GA-FD-T20, MD-FD-T20, MD-FD-T80) and a control without AITC addition. The second trial included four soups with different spray-dried AITC formulations (GA-SD, GA-SD-T20, MD-SD-T20, MD-SD-T80) and a control without AITC. Non-encapsulated AITC was not included in the soup, as the researchers have evaluated it in a past study [25]. Both sensory trials followed the procedure outlined below.

#### 2.2.1. Participants

This study was approved by the Acadia University Research Ethics Board (REB#13-72), followed the ethical standards outlined in the Declaration of Helsinki (2013), and all participants gave informed consent before the testing. Inclusion in the first sensory trial did not preclude participation in the second sensory trial. All participants declared that they were not currently breastfeeding or pregnant, had no known smell or taste defects, and were non-smokers. The participants also declared that they had not tested positive for COVID in the past month, were not allergic to any of the ingredients, and did not have any conditions or consume any medications that altered their oral functioning (e.g., related to the senses, salivation, mastication, swallowing, or dentition). The first trial included 93 participants (58 female, 40 male, and 2 other; average age = 38), and the second trial included 79 participants (37 female, 40 male, and 2 other; average age = 37).

#### 2.2.2. Samples

Commercially available low-sodium tomato soup (Campbell Company of Canada; Mississauga, ON, Canada; ingredients include water, tomato paste, wheat flour, sugar, salt, yeast extract, flavour, ascorbic acid, citric acid) was placed in crock pots the night before the sensory trial. The selected encapsulated AITC powders were added to individual batches of soup in 0.500 mg/100 mL (selected amount based on GC-FID results). Soups were stirred for 90 s and stored at 4 °C overnight. Soups were evaluated by research assistants (*n* = 8) employed in the sensory lab to validate the amount of encapsulated AITC added to the soup formulations. During the sensory testing, the soups were warmed over medium heat. Samples of 20 mL were served in clear 30 mL plastic cups (at approximately 65 °C) and were blinded with three-digit codes. The samples were presented one at a time in a randomized order.

#### 2.2.3. Sensory Evaluation

The testing occurred in individual sensory booths in the Centre for the Sensory Research of Food at Acadia University (Wolfville, NS, Canada), and the questionnaire was presented using Compusense software (Version Version 25.0.33857, Compusense, Guelph, ON, Canada) on iPads.

In each sensory trial, the participant evaluated five samples. The questionnaire prompted consumers to consume some of the sample and evaluate it using a nine-point hedonic scale where 1 = Dislike Extremely and 9 = Like Extremely regarding overall liking, flavour, and texture/mouthfeel. The participants then completed a rate-all-that-apply (RATA) [37] question that included the attributes: salty, tomato flavour, sweet, bitter, sour, spicy, thick, creamy, metallic, and savoury. These attributes were selected based on preliminary sensory testing with research assistants familiar with sensory evaluation and a literature review [38,39,40,41,42,43]. Participants were instructed to select all attributes they perceived in the sample and rate them from 1 = Extremely Low to 7 = Extremely High. The attributes were presented in a randomized order, and attributes that were not selected were assigned a numerical value of 0. A 30 s break was initiated between samples, where participants were instructed to rinse their mouths with water. The participants also completed demographic questions (i.e., age and gender).

### 2.3. Statistical Analysis

#### 2.3.1. Chemical Analysis

Statistical analyses were performed using R studio (Version 2024.04.2+764) to evaluate differences in the encapsulated AITC based on the encapsulation processes and the different formulations used. Results were analyzed using linear mixed-effects regression (lmer) with replication as a random factor to quantify surface oil, encapsulation efficiency, and moisture content. These tests were followed by multiple comparisons of means (Tukey’s HSD test) using the “emmeas” package.

#### 2.3.2. Sensory Evaluation

The results of both sensory trials were analyzed as outlined below to evaluate if the encapsulated AITC impacted the consumers’ sensory perception and the acceptability of the soup. Each sample’s mean and standard deviation were calculated, and the hedonic values were evaluated using a two-way ANOVA and Tukey’s HSD test (95% confidence interval). The RATA data were treated as continuous and interpreted as an eight-point scale considered ‘not applicable’ as intensity = 0 [44]. The mean and standard deviation for each attribute per sample were calculated. A linear mixed-effects model was performed for the intensities of each sensory attribute to evaluate if significant differences exist across the different samples. The participant was a random effect, and the sample was fixed. If significant differences exist, Tukey’s HSD test (at a 95% confidence interval) was performed [44,45]. A *t*-test was used to compare the results of the encapsulated formulations created with different drying methods (spray-dried vs. freeze-dried). Demographic information was evaluated using descriptive statistics. Analyses were performed in XLSTAT (Lumivero; Denver, CO, USA) in Microsoft Excel.

## 3. Results and Discussion

### 3.1. Physico-Chemical Characteristics

#### 3.1.1. Encapsulation Efficiency

The encapsulation efficiency (EE) as mg of AITC/g of powder is reported in Table 1 (and Figure S2). The EE expressed as % of AITC encapsulated in 0.5 g of powder is reported in Table S1 and Figure S3 (Supplementary Materials). Very few studies have described the encapsulation of sulphur aroma compounds through spray-drying using gum Arabic or maltodextrin. Uekane et al. [46] performed the encapsulation of 2-furfurylthiol using both gum Arabic and maltodextrin, obtaining good retention of the active ingredient with 67% EE. The encapsulation of wasabi flavour, rich in sulphur compounds such as AITC, was performed using maltodextrin in combination with maize starch, providing an EE in the range of 40–50 mg of AITC/100 g of sample [47] and a constant release of wasabi flavour over storage conditions. The preparation of AITC microcapsules through freeze-drying was described by Jin et al. [48], using chitosan as wall material and a cross-linking agent (glutaraldehyde). The EE% of AITC in the microcapsules was dependent on the homogenization time and the ratio of core material to emulsifier, reaching 96% under optimal conditions.

In our study, FD resulted in consistently low AITC retention, and no significant differences were observed between FD samples with and without surfactants. Similar trends were observed across both wall materials in the FD samples with EE ranging from 3.40 ± 0.13 mg AITC/g of powder (0.67 ± 0.02% EE) (MD-FD) to 13.36 ± 1.33 mg AITC/g of powder (2.64 ± 0.26% EE) (MD-FD-T20) (*p* > 0.05) using MD as wall material and from 5.58 ± 0.71 mg AITC/g of powder (1.10 ± 0.14% EE) (GA-FD-T80) to 13.97 ± 0.27 mg AITC/g of powder (2.76 ± 0.05% EE) (GA-FD) (*p* > 0.05) with GA as wall material. While extremely low AITC retention was observed in SD samples without surfactants −0.90 ± 0.1 mg AITC/g of powder for GA-SD (0.18 ± 0.001% EE) and 0.60 ± 0.1 mg AITC/g of powder for MD-SD (0.12 ± 0.003% EE), the addition of surfactants significantly improved the AITC retention (*p* < 0.001, except for MD-SD-T20 where *p* = 0.912). The highest retention of AITC was observed in the GA-SD-T20 formulation (136.71 ± 4.32 mg AITC/g of powder) (26.99 ± 0.85% EE).

The high thermal stress associated with SD makes heat-sensitive compounds like AITC more likely to degrade during encapsulation than FD [49,50], though the difference observed between methods is marginal under some conditions [51]. Furthermore, Chen et al. [52] observed greater losses of limonene volatiles in FD samples than in SD samples, noting that while volatiles may be lost in both methods due to environmental evaporation, FD matrices may facilitate greater losses due to their less robust structure, which allows for greater diffusion between the core and wall material during processing and storing. Yaman et al. [53] also achieved greater retention of volatiles using the SD method compared to the FD method. While the heat conditions used in SD may impact the volatile retention rate, the greater emulsion stability achieved by using the surfactant has previously demonstrated the ability to protect volatile compounds during SD [54]. Under certain conditions, formulations resulted in relatively high AITC retention using either surfactant (i.e., T20 or T80), indicating that both may be suitable for encapsulating AITC via SD. T80 differs from T20 primarily due to its longer hydrocarbon tail, which increases its hydrophobicity. The longer hydrophobic chain enhances the packing density at the oil-water interface, forming more stable micelles and potentially improving the encapsulation efficiency [55]. Both T20 and T80 increased the retention of AITC when SD with GA (*p* < 0.001), although only T80 significantly increased the retention of AITC when SD with MD (*p* < 0.001). Past studies have identified T20 as a suitable surfactant for AITC [29,34] but this study showed that T80 increased the retention of AITC during SD, agreeing with the study by [30].

#### 3.1.2. Surface Oil Percentage

The surface oil percentage change is presented in Table 2 and Figure S4. The consistently high percentage change observed indicates a substantial loss of AITC during the rinsing process. This result suggests that a significant proportion of the AITC is present on the surface of the microcapsules, or that the microcapsules may have surface cracks, allowing AITC to leak from the matrix (Rajabi et al. 2015) [56]. However, it is also possible that the rinsing process was too harsh and damaged the structural integrity of the microcapsules, leading to inflated percentages.

While most of the differences in formulations were insignificant (*p* > 0.05), a few trends can still be observed. In general, the powders that used GA as wall material tended to have slightly lower surface oil percentages than those that used MD as wall material. This result suggests that GA might form a somewhat more durable encapsulation matrix than MD when used in this context. This observation aligns with the properties of GA, which include a strong film-forming ability that helps it create a strong barrier around the encapsulated product [57]. The use of GA presents some limitations, including its low glass transition temperature, which may impact the quality of the product [58]. MDs are commonly used in spray-drying due to their high molecular weight, high glass transition temperature, high solubility, and low viscosity, which provide several benefits during the spray-drying process, including strength, stability, and re-solubilization [58]. However, when used alone, MDs lack a strong film-forming ability, which can hinder their efficiency in preserving sensitive compounds [58].

FD samples also tended to have slightly lower surface oil percentages than SD samples, indicating that the FD process may better retain AITC within the matrix. This finding is consistent with a previous study that identified FD as being better suited to sensitive compounds due to its gentler processing conditions [59]. This effect may also be due to the lower retention of AITC observed in FD powders (Table 1), which contributes to less significant losses during rinsing. The use of surfactant did not significantly impact the surface oil percentages (*p* < 0.05), except for GA-FD-T80, which had significantly lower surface oil percentages compared to other GA-FD samples (*p* < 0.05). The surfactant concentration may have been too low to alter the encapsulation stability, or the drying method might have dominated the surface oil outcome more than the surface presence. In the case of GA-FD-T80, synergistic emulsification may have occurred, as the combination of gum Arabic and T80 may have led to a more stable and uniform emulsion. This could have resulted in better encapsulation of the oil. In addition, FD involves slower water removal than SD, leading to a stronger, more cohesive matrix formation, especially when both GA and T80 are present [60].

#### 3.1.3. Moisture Content

The moisture contents of the powders are presented in Table 3. FD samples displayed greater moisture retention after encapsulation compared to the SD samples. This trend is consistent with other studies that report higher moisture content in FD powders than in SD powders [61,62]. Pudziuvelyte et al. [62] attributed this result to the lower temperature conditions of FD, which can result in greater residual moisture as it produces smaller pore sizes that may prevent water from evaporating. Additionally, FD powders tend to be more hygroscopic and may absorb more environmental moisture [62]. The addition of surfactants did not consistently result in clear trends across all conditions; however, surfactants tended to increase the moisture content of the samples, particularly when T20 was used. T20 has a greater hydrophile-lipophile balance value than T80, indicating it is more hydrophilic [63]. This property of T20 may enhance its attraction to water molecules and, therefore, moisture retention in the powder matrix. The FD samples made with GA and T20 had the highest moisture content, significantly different from the formulation without surfactant (*p* < 0.05).

GA and MD powders were tested individually, and both demonstrated similar changes in moisture content. Although not significant, GA exhibited a slightly higher moisture content compared to MD. This trend is consistent across the samples tested. Although both are polysaccharides, GA contains more hydroxyl groups than MD, which enhances its hydrophilicity [64] and, therefore, may increase its interactions with water. Furthermore, a previous study reported that MD was associated with decreased hygroscopicity compared to GA [65], suggesting that powders prepared with GA may absorb more water from their environment.

#### 3.1.4. Scanning Electron Microscopy

**Figure 1:**
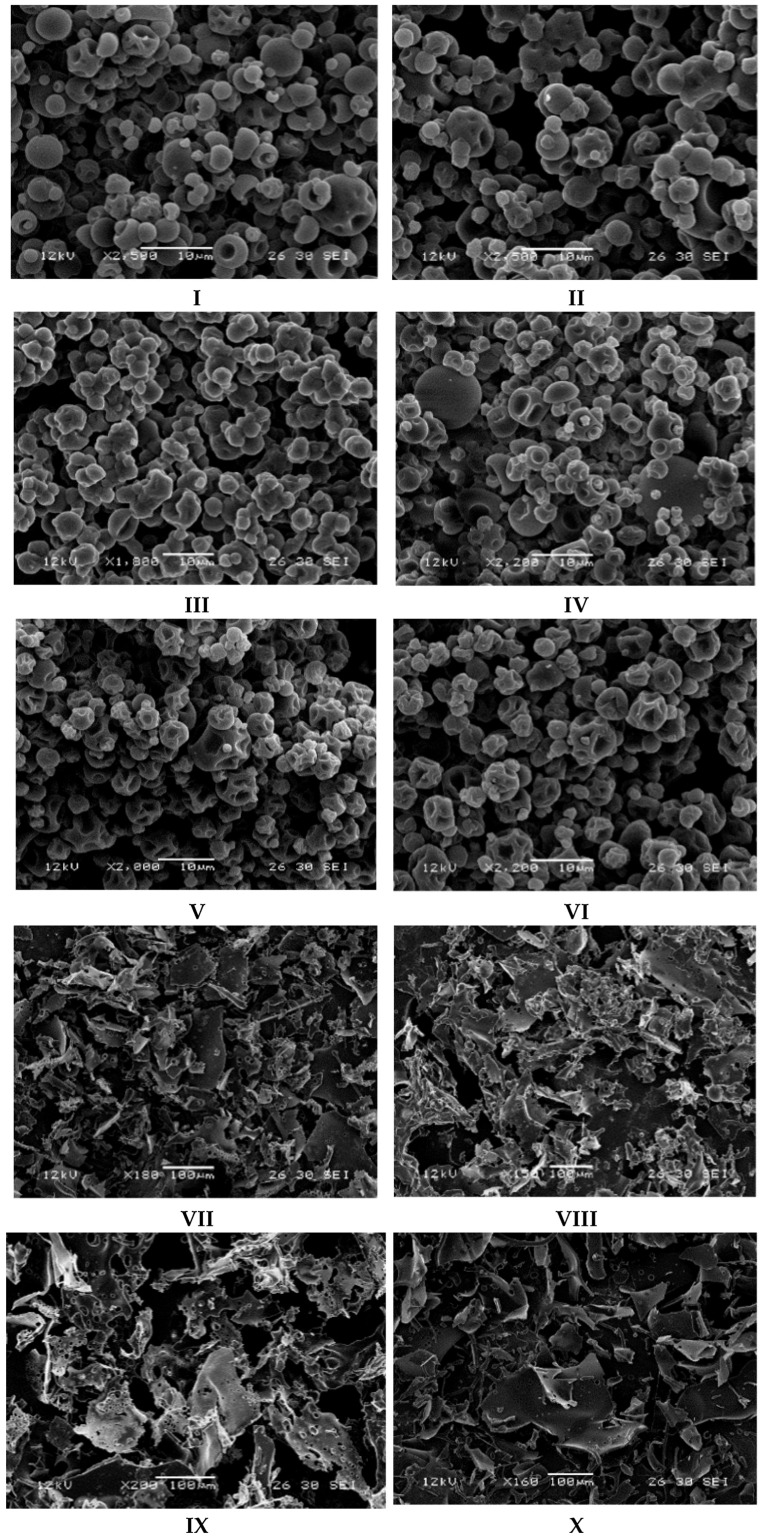

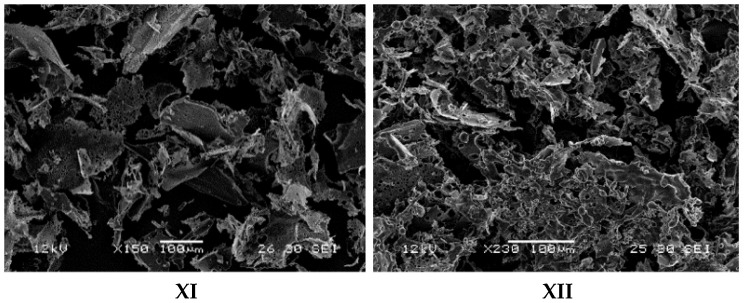
SEM Images of allyl isothiocyanate (AITC) microcapsules produced using freeze-drying (FD) and spray-drying (SD) with different formulations. Encapsulating agents include gum Arabic (GA) and maltodextrin (MD), with emulsifiers Tween-20 (T20) and Tween-80 (T80). (**I**) = GA-SD; (**II**) = MD-SD; (**III**) = GA-SD-T20; (**IV**) = MD-SD-T20; (**V**) = GA-SD-T80; (**VI**) = MD-SD-T80; (**VII**) = GA-FD; (**VIII**) = MD-FD; (**IX**) = GA-FD-T20; (**X**) = MD-FD-T20; (**XI**) = GA-FD-T80; (**XII**) = MD-FD-T80.

Microencapsulated powders were photographed using a scanning electron microscope (Figure 1). SD microcapsules without surfactants exhibit a similar surface morphology, characterized by smooth surfaces and moderate surface indentation. Microcapsules using GA wall material and no surfactant have a more donut-like shape, with one-centred surface depression, compared to the no-surfactant MD microcapsules with a more irregular indentation. MD SD microcapsules with T20 exhibit a combination of donut-like and irregular surface indentation. Both GA and MD microcapsules with T80 appear to have the most irregular surface indentation, exhibiting a shape that more closely resembles a truncated octahedron rather than a sphere. Hollow surfaces are a common characteristic of SD microcapsules, often resulting from the evaporation and rupture of trapped residual solvent through the outer shell [66]. Typically, surfactants with higher molecular weights, such as T20 and T80, produce microcapsules with greater surface irregularity due to their stabilizing ability. The higher-weight surfactants tend to accumulate at the surface of microcapsules, and due to this behaviour during the drying process, they tend to exhibit more complex surface features [67]. Less hollow dentation can generally be observed in SD microcapsules with surfactants, possibly because of the influence of surfactants on the encapsulated powders [68].

Some particle agglomeration can be observed in all the SD microcapsules due to the proximity of dried particles to the drying chamber. Small dry particles can re-enter the drying chamber, thereby facilitating the formation of agglomerates [69]. SD microcapsules exhibit similar size heterogeneity, with particle sizes ranging from approximately 1 µm to 10 µm in diameter.

FD microcapsules exhibited similar surface morphology and size distribution. Holes and gaps can be observed in all microcapsules, along with sharp edges and irregular, flat shapes. The porous, jagged structure is characteristic of FD, as they are believed to develop from fine ice crystals that evaporate during the sublimation process [70].

### 3.2. Sensory Evaluation

Tomato soups with added encapsulated AITC led to consistently low consumer-perceived saltiness levels across all samples (Table 4 and Table 5). The control (without AITC) had the lowest mean perceived saltiness across both sensory trials; however, no significant differences were observed between control soup and encapsulated AITC samples (*p* > 0.05). This lack of significant saltiness enhancement contrasts with findings by Amyoony et al. [25]. However, the past study evaluated soup with unencapsulated AITC added at its detection threshold. In our study, the encapsulation of the AITC may have impacted its detection threshold, and future studies should investigate the detection threshold of the different encapsulated AITC formulations. The results indicate that the encapsulation process impacted AITC’s cross-modal interaction on saltiness perception.

Encapsulated AITC also did not significantly influence the soups’ sweet, bitter, sour, or savoury (umami) tastes compared to the control (*p* > 0.05). Previous studies have shown that oral irritants mask other flavours [71,72]. This masking effect may result from dominant sensory inputs or competition at receptor sites, diminishing the perception of other sensations [71]. Amyoony et al. [25] found that unencapsulated AITC suppressed sweetness and savouriness while imparting bitterness; this effect was not observed in the present study. AITC has been shown to activate TAS2R38-PAV receptors, which are associated with a bitter taste in most people [73]. Encapsulation may be able to mask this bitterness [74]; this could explain the differences in findings.

Further investigation into the precise release profile of the encapsulated powders is necessary to determine whether they fully dissolved in the soup’s aqueous medium or if gradual release could have continued in the mouth upon contact with saliva. If fully dissolved, other effects, such as wall material composition or surfactants, may have influenced the perception of AITC. Conversely, if the encapsulation matrix remained partially intact during consumption, it could have hindered AITC interaction with receptors or limited volatile release, impacting its flavour perception. Dang et al. [75] studied the release kinetics of encapsulated acerola using MD and GD wall materials. Most bioactive components were diffused into their surrounding aqueous medium (water, 10% ethanol, or 3% acetic acid) within ten m, characteristic of most microcapsules. This suggests that most of the AITC likely diffused into the surrounding soup before consumption; however, surfactants and the encapsulation method may impact the maximum diffusion.

The addition of AITC to the soup did not significantly increase its perceived spiciness compared to the control (Table 5), regardless of the encapsulation method (*p* > 0.05). The mean spiciness rating for all soups was between 0 and 1, indicating that it was quite low. AITC seemed to have a greater influence on the perceived tomato flavour in the soups containing FD AITC rather than SD AITC. Soup MD-FD-T80 significantly differed from the control (*p* < 0.05), and the remaining FD soups (soups GA-FD, GA-FD-T20, and MD-FD-T20) were statistically similar to both the control and the MD-FD-T80 soup. Differences between the control and the MD-FD-T20 for perceived tomato flavour may be related to the role of volatile organic compounds (VOCs) in shaping perception through complex multisensory interactions [76]. VOCs, such as those found in AITC, are known to influence gustatory, olfactory, and trigeminal perceptions, significantly affecting how consumers experience flavour. Given that much of what the consumer perceives as taste is derived from olfactory cues [76], the volatile nature of AITC may have enhanced the overall aroma of the soup, contributing to a heightened perception of tomato flavour. However, further investigation is necessary as only the MD-FD-T80 sample was significantly different than the control.

The encapsulation method impacted the soups’ perceived thickness (Table 5). While significant differences were not observed between the control and SD-AITC soups, differences were observed in the FD soups. The GA-FD sample was significantly higher in thickness than all other soups except for the MD-FD-T80 sample (*p* < 0.05). The creaminess of the GA-FD sample was also significantly higher than the control and MD-FD-T20 samples (*p* > 0.05). A study by Lyu et al. (2021) [77] found that the oral burn sensation caused by CP in soup increased thickness discrimination thresholds, attributing the effect to cross-modality impacts, increased neural noise, or redirection of attentional focus. All soups contributed to low metallic flavour perception, with the control soups being statistically like those containing encapsulated AITC. This finding contrasts with previous observations by Amyoony et al. [25], who reported increased metallic sensations in low-sodium soups containing AITC. Amyoony et al. [25] highlighted that the increased metallic sensations might result from consumers relating bitterness and metallic flavour in their evaluations. Increased perceptions of bitterness in some individuals have been associated with other chemical irritants, including capsaicin, piperine, and zingerone, potentially due to their ability to stimulate bitterness receptors [78]. It is promising that the encapsulated AITC was not associated with increased bitterness or metallic flavours in this study.

The participants’ liking scores in this study are presented in Table 6. The soups with SD AITC did not significantly differ from the SD control, suggesting that the encapsulated AITC did not noticeably alter the soup’s overall liking, flavour, or texture. This finding follows the previous observations, where the SD AITC soups did not significantly alter the soup’s basic tastes or other sensory qualities. The addition of FD AITC seemed to have more effect on the soups’ overall liking, flavour, and texture, especially in the MD-FD-T80 soup. Previous studies have demonstrated an association between chemical irritants and perceived mouthfeel (i.e., physical and textural sensations) [79,80]. MD-FD-T80 soup was also associated with increased perceived thickness (*p* < 0.05) and creaminess (*p* < 0.05) compared to the control, which may have influenced its textural properties. This increase in perceived thickness and creaminess could be attributed to cross-modal interactions and increased neural noise [77], as discussed above. Also, a past study found that capsaicin, another chemical irritant, increased saliva secretion, which in turn impacted textural perception [81].

Encapsulated AITC that had the same wall material and surfactant but was made using different methods was compared (i.e., GA-SD was compared to GA-FD). However, no significant differences were identified (*p* > 0.05). Based on this study, it appears that FD soups, and most notably MD-FD-T80, may be associated with desirable sensory qualities for consumers. It is essential to understand consumer perception of food products, as this informs the development of products that are acceptable to consumers and, in turn, are likely to be purchased by them. However, consumers or untrained panellists are not always able to identify small differences between food items, so trained panellists and instrumental aroma analysis can be used. Both trained panellists and instrumental aroma analysis can detect minor differences between the formulations. These evaluations, in conjunction with consumer feedback, can help assess how these minor differences affect consumer acceptability.

Determining the most consumer-accepted encapsulation method in food products requires experimental evaluation, as no universally preferred approach exists. Increased solubility could be advantageous for maximizing AITC flavour release. However, higher concentrations of AITC are associated with unfavourable flavours [25]. Therefore, the potentially lower solubility of FD powders may have masked some of the AITC flavour by retaining it more effectively within the matrix, contributing to its greater liking. Future studies should investigate whether FD AITC powders, at a higher concentration, may enhance saltiness without imparting off-flavour. SD is the most often used drying method in the food industry due to its speed, cost-effectiveness, and ability to operate continuously [82]. In contrast, FD is significantly more expensive, time-consuming, and can be logistically more complex. As stated above, MD-FD-T80, a freeze-dried encapsulation was preferred by consumers, but the expense of freeze-drying may make it impractical from an industrial application point of view. Furthermore, it is also essential to highlight the greater moisture content observed in the FD powders. The higher water activity may have contributed to increased losses of AITC during storage, potentially reducing its concentration in sensory trials. Once again, this may not make it feasible in industrial setting to use FD AITC formulations. Future studies should investigate the short-term and long-term stability of these powders to assess their shelf life. Additionally, the increased water activity in FD powders may promote microbial growth, affecting their quality, safety, and shelf life [83]. Future studies should characterize the release kinetics of encapsulated AITC in various food matrices, including the soup matrix used in this study. Future research could also investigate how the addition of encapsulated AITC to the soup affects salivary flow and sodium release. Further research is needed on encapsulated AITC to evaluate its suitability and effectiveness in food products.

## 4. Conclusions

This study evaluated various methods for AITC encapsulation and examined the effects on physicochemical characteristics and sensory properties. Among the methods tested, FD resulted in relatively low AITC retention, which was not significantly enhanced with surfactants. SD without surfactants resulted in very low AITC retention. The SD with surfactants led to higher AITC retention than FD. SEM analysis revealed significant morphological differences between the encapsulated products produced by the two drying methods. SD produced small, smooth, and spherical microcapsules, while FD produced larger, rigid, and porous ones. The encapsulated AITC addition to the soup did not increase saltiness perception and negatively impacted the cross-modal interactions identified in past studies on AITC; however, the soups were not associated with bitterness and metallic flavours (common off-flavours of AITC). Future research is needed to evaluate its specific release behaviour, overall stability, and shelf life. However, the encapsulation efficiency of both the FD and SD methods used in this study are quite low and would not be feasible in an industrial setting. Future studies need to continue to evaluate new methods of encapsulation for AITC.

Also, future research is needed to evaluate its specific release behaviour, overall stability, and shelf life, as well as to establish whether encapsulated AITC can be a viable additive to reduce the sodium content of foods. Many more studies are needed to evaluate the cross-modal interactions of chemical irritants and how they can be used to create acceptable food products. Future studies should utilize trained panellists and dynamic sensory methodologies to further characterize encapsulated AITC. Furthermore, future research should explore the application of encapsulated AITC in solid food matrices, which may impact its sensory characteristics.

## Figures and Tables

**Table 1:** Encapsulation efficiency of allyl isothiocyanate (AITC) microcapsules (mg AITC/g powder) ± SE produced using freeze-drying (FD) and spray-drying (SD) with different formulations. Encapsulating agents include gum Arabic (GA) and maltodextrin (MD), with emulsifiers Tween-20 (T20) and Tween-80 (T80). Groups containing significant differences are marked with an asterisk (*p* < 0.05; *n* = 3; Tukey’s test).

Wall Material	Encapsulation Method	Emulsifier	EE mg AITC/g Powder	±SE	z	*p*
GA	FD	None	13.97	0.27	-	-
GA	FD	T20	7.12	2.16	−0.932	0.999
GA	FD	T80	5.58	0.71	−1.141	0.993
GA	SD	None	0.90	0.01	-	-
GA	SD	T20	136.71	4.32	18.483	<0.001 *
GA	SD	T80	76.71	2.83	10.317	<0.001 *
MD	FD	None	3.40	0.13	-	-
MD	FD	T20	13.36	1.33	1.354	0.972
MD	FD	T80	8.44	0.47	0.685	1.000
MD	SD	None	0.60	0.01	-	-
MD	SD	T20	12.31	0.76	1.493	0.912
MD	SD	T80	85.32	17.01	11.529	<0.001 *

**Table 2:** Percentage surface oil of allyl isothiocyanate (AITC) microcapsules (%) ± SE produced using freeze-drying (FD) and spray-drying (SD) with different formulations. Encapsulating agents include gum Arabic (GA) and maltodextrin (MD), with emulsifiers Tween-20 (T20) and Tween-80 (T80). Groups containing significant differences are marked with an asterisk (*p* < 0.05; *n* = 3; Tukey’s test).

Wall Material	Encapsulation Method	Emulsifier	% Surface Oil	±SE	z	*p*
GA	FD	None	83.5	2.43	-	-
GA	FD	T20	89.3	1.77	2.887	0.1451
GA	FD	T80	94.0	2.02	5.258	<0.01 *
GA	SD	None	84.9	0.838	-	-
GA	SD	T20	87.8	0.794	1.468	0.9493
GA	SD	T80	90.3	1.11	2.695	0.2286
MD	FD	None	89.5	1.30	-	-
MD	FD	T20	94.6	0.680	2.573	0.2951
MD	FD	T80	90.2	1.57	0.338	1
MD	SD	None	92.8	1.32	-	-
MD	SD	T20	89.3	1.14	−1.771	0.8341
MD	SD	T80	89.3	0.602	−1.767	0.836

**Table 3:** Mean moisture content of allyl isothiocyanate (AITC) microcapsules (%) ± SE produced using freeze-drying (FD) and spray-drying (SD) with different formulations. Encapsulating agents include gum Arabic (GA) and maltodextrin (MD), with emulsifiers Tween-20 (T20) and Tween-80 (T80). Groups containing significant differences are marked with an asterisk (*p* < 0.05; *n* = 3; Tukey’s test).

Wall Material	Encapsulation Method	Surfactant	% Change in Moisture Content	±SE	z	*p*
GA	None	None	8.27	0.177	-	-
MD	None	None	4.71	0.874	−1.45	0.977
GA	FD	None	5.35	1.34	-	-
GA	FD	T20	24.4	3.80	7.74	<0.01 *
GA	FD	T80	11.5	3.23	2.50	0.409
GA	SD	None	6.96	0.600	-	-
GA	SD	T20	4.15	1.793	−1.14	0.997
GA	SD	T80	0.696	0.0569	−2.50	0.379
MD	FD	None	5.05	0.581	-	-
MD	FD	T20	2.56	1.74	−1.01	0.999
MD	FD	T80	6.78	2.72	0.700	1
MD	SD	None	1.07	0.251	-	-
MD	SD	T20	6.37	0.567	2.15	0.666
MD	SD	T80	4.45	0.487	1.37	0.985

**Table 4:** Consumer scores for the basic tastes (±standard deviation) of the soup samples, including AITC produced using freeze-drying (FD) and spray-drying (SD) with different formulations. Encapsulating agents include gum Arabic (GA) and maltodextrin (MD), with emulsifiers Tween-20 (T20) and Tween-80 (T80).

Sample	Salty	Sweet	Bitter	Sour	Savoury
*Spray Dried Sensory Trial (n = 79)*					
Control	2.7 _a_^1,2^ ± 1.1	3.6 _a_ ± 1.1	0.9 _a_ ± 0.6	1.5 _a_ ± 0.7	3.1 _a_ ± 1.2
GA-SD	3.1 _a_ ± 1.2	3.5 _a_ ± 1.0	1.2 _a_ ± 1.0	1.3 _a_ ± 0.7	3.0 _a_ ± 1.0
GA-SD-T20	2.9 _a_ ± 1.0	3.5 _a_ ± 1.2	1.1 _a_ ± 0.6	1.5 _a_ ± 0.8	2.9 _a_ ± 1.1
MD-SD-T20	2.8 _a_ ± 1.3	3.8 _a_ ± 1.0	1.1 _a_ ± 0.7	1.4 _a_ ± 1.0	3.2 _a_ ± 1.2
MD-SD-T80	2.9 _a_ ± 1.4	3.7 _a_ ± 1.4	1.2 _a_ ± 0.6	1.4 _a_ ± 0.4	3.0 _a_ ± 1.0
*Freeze-Dried Sensory Trial (n = 93)*					
Control	2.8 _a_ ± 1.3	3.5 _a_ ± 1.7	1.1 _a_ ± 1.4	1.4 _a_ ± 0.8	2.6 _a_ ± 1.1
GA-FD	3.3 _a_ ± 1.5	3.5 _a_ ± 1.6	1.2 _a_ ± 1.6	1.6 _a_ ± 0.6	2.9 _a_ ± 1.2
GA-FD-T20	3.2 _a_ ± 1.4	3.4 _a_ ± 1.0	1.1 _a_ ± 1.5	1.5 _a_ ± 0.7	2.8 _a_ ± 1.0
MD-FD-T20	3.0 _a_ ± 1.4	3.2 _a_ ± 1.4	0.9 _a_ ± 1.0	1.5 _a_ ± 0.7	2.7 _a_ ± 1.6
MD-FD-T80	3.2 _a_ ± 1.5	3.6 _a_ ± 1.4	1.2 _a_ ± 1.0	1.6 _a_ ± 0.7	3.2 _a_ ± 1.4

^1^ Data collection on a nine-point hedonic scale (1 = Extremely Low to 7 = Extremely High). ^2^ Means in the same column with the same letter (within the same trial), are not significantly different (*p*  >  0.05).

**Table 5:** Consumer scores for the other sensory properties (±standard deviation) of the soup samples including AITC, produced using freeze-drying (FD) and spray-drying (SD) with different formulations. Encapsulating agents include gum Arabic (GA) and maltodextrin (MD), with emulsifiers Tween-20 (T20) and Tween-80 (T80).

Sample	Spicy	Tomato Flavour	Thick	Creamy	Metallic
*Spray Dried Sensory Trial (n = 79)*					
Control	0.5 _a_^1,2^ ± 0.4	4.8 _a_ ± 1.5	3.9 _a_ ± 1.2	3.1 _a_ ± 1.0	1.1 _a_ ± 1.0
GA-SD	0.5 _a_ ± 0.5	4.7 _a_ ± 1.3	3.0 _a_ ± 1.2	2.7 _a_ ± 1.6	1.2 _a_ ± 1.2
GA-SD-T20	0.5 _a_ ± 0.2	4.4 _a_ ± 1.4	3.4 _a_ ± 1.4	2.9 _a_ ± 1.7	1.2 _a_ ± 1.2
MD-SD-T20	0.4 _a_ ± 0.6	4.8 _a_ ± 1.6	3.7 _a_ ± 1.5	3.3 _a_ ± 1.2	1.0 _a_ ± 1.1
MD-SD-T80	0.5 _a_ ± 0.4	4.5 _a_ ± 1.0	3.0 _a_ ± 1.0	2.7 _a_ ± 1.0	1.1 _a_ ± 1.3
*Freeze-Dried Sensory Trial (n = 93)*					
Control	0.7 _a_ ± 0.2	3.7 _a_ ± 1.1	2.6 _c_ ± 1.1	2.4 _b_ ±1.0	1.2 _a_ ± 1.2
GA-FD	0.9 _a_ ± 0.3	4.5 _ab_ ± 1.6	4.1 _ab_ ± 1.7	3.1 _a_ ± 1.4	1.1 _a_ ± 0.8
GA-FD-T20	0.8 _a_ ± 0.5	4.3 _ab_ ± 1.5	3.2 _c_ ± 1.3	2.7 _ab_ ± 1.5	1.0 _a_ ± 0.7
MD-FD-T20	0.7 _a_ ± 0.5	4.1 _ab_ ± 1.4	2.6 _c_ ± 1.3	2.2 _b_ ± 1.6	1.0 _a_ ± 0.5
MD-FD-T80	0.9 _a_ ± 0.2	4.7 _b_ ± 1.3	3.6 _bc_ ± 1.3	3.0 _ab_ ± 1.2	0.9 _a_ ± 0.7

^1^ Data collection on a nine-point hedonic scale (1 = Extremely Low to 7 = Extremely High). ^2^ Means in the same column with the same letter (within the same trial), are not significantly different (*p*  >  0.05).

**Table 6:** Consumer mean liking scores (±standard deviation) for flavour, texture, and overall liking of the soup samples including AITC produced using freeze-drying (FD) and spray-drying (SD) with different formulations. Encapsulating agents include gum Arabic (GA) and maltodextrin (MD), with emulsifiers Tween-20 (T20) and Tween-80 (T80).

Sample	Overall Liking	Flavour	Texture
*Spray Dried Sensory Trial (n = 79)*			
Control	6.4 _a_^1,2^ ± 1.4	6.4 _a_ ± 1.0	6.3 _a_ ± 1.9
GA-SD	6.2 _a_ ± 1.3	6.1 _a_ ± 1.2	6.4 _a_ ± 2.0
GA-SD-T20	5.8 _a_ ± 1.4	5.8 _a_ ± 1.6	6.2 _a_ ± 2.1
MD-SD-T20	6.2 _a_ ± 1.6	6.3 _a_ ± 1.9	6.6 _a_ ± 1.7
MD-SD-T80	6.2 _a_ ± 1.1	6.3 _a_ ± 2.0	6.7 _a_ ± 1.8
*Freeze-Dried Sensory Trial (n = 93)*			
Control	5.5 _b_ ± 1.4	5.5 _b_ ± 1.4	5.6 _b_ ± 1.9
GA-FD	5.9 _ab_ ± 1.4	6.0 _ab_ ± 1.4	5.9 _ab_ ± 1.9
GA-FD-T20	5.7 _b_ ± 1.9	5.8 _b_ ± 1.3	5.9 _ab_ ± 1.4
MD-FD-T20	5.8 _b_ ± 2.1	5.7 _b_ ± 1.5	6.0 _ab_ ± 1.3
MD-FD-T80	6.6 _a_ ± 1.9	6.6 _a_ ± 1.6	6.6 _a_ ± 1.4

^1^ Data collection on a nine-point hedonic scale (1 = Dislike Extremely and 9 = Like Extremely). ^2^ Means in the same column with the same letter (within the same trial), are not significantly different (*p*  >  0.05).

## Data Availability

The original contributions presented in this study are included in the article/Supplementary Materials. Further inquiries can be directed to the corresponding author.

## Supplementary Materials

The following supporting information can be downloaded at: https://www.mdpi.com/article/10.3390/foods14223810/s1, Table S1. Encapsulation efficiency of allyl isothiocyanate (AITC) microcapsules (%) ± SE produced using freeze-drying (FD) and spray-drying (SD) with different formulations. Encapsulating agents include gum Arabic (GA) and maltodextrin (MD), with emulsifiers Tween-20 (T20) and Tween-80 (T80). Groups containing significant differences are marked with an asterisk (*p* < 0.05; *n* = 3; Tukey’s test). Figure S1: Calibration curve for allyl isothiocyanate (AITC) at concentrations ranging from 31.25 to 3000 ng/µL, spiked with 25 µL of 4960 ng/µL phenyl isothiocyanate (PITC) internal standard (IS). Data was acquired performing Gas Chromatography–Flame Ionization Detector (GC-FID) analysis and the curve was generated by plotting the peak area ratios of AITC to PITC against the standard AITC concentrations prepared via serial dilutions. Figure S2. Summary of the mean (*n* = 3) encapsulation efficiency of allyl isothiocyanate (AITC) microcapsules (mg AITC/g powder) ± SE produced using freeze-drying (FD) and spray-drying (SD) with different formulations. Encapsulating agents include gum Arabic (GA) and maltodextrin (MD), with emulsifiers Tween-20 (T20) and Tween-80 (T80). Figure S3: Summary of the mean (*n* = 3) encapsulation efficiency of allyl isothiocyanate (AITC) microcapsules (%) ± SE produced using freeze-drying (FD) and spray-drying (SD) with different formulations. Encapsulating agents include gum Arabic (GA) and maltodextrin (MD), with emulsifiers Tween-20 (T20) and Tween-80 (T80); Figure S4. Summary of the mean (*n* = 3) percentage surface oil of allyl isothiocyanate (AITC) microcapsules (%) ± SE produced using freeze-drying (FD) and spray-drying (SD) with different formulations. Encapsulating agents include gum Arabic (GA) and maltodextrin (MD), with emulsifiers Tween-20 (T20) and Tween-80 (T80).
