# Supplementary material for: Encapsulation of Allyl Isothiocyanate by Freeze- and Spray-Drying: Effects on Retention and Sensory Perception in Sodium-Reduced Soups
Source: Foods. 2025 Nov 7;14(22):3810. doi: 10.3390/foods14223810 (PMC12651518; doi:10.3390/foods14223810)
Supplement: Supplementary file 1 [file foods-14-03810-s001.zip › foods-3944293-supplementary.pdf]

Table S1. Encapsulation efficiency of allyl isothiocyanate (AITC) microcapsules (%) ± SE produced using freeze-drying (FD) and spray-drying (SD) with different formulations. Encapsulating agents include gum Arabic (GA) and maltodextrin (MD), with emulsifiers Tween-20 (T20) and Tween-80 (T80). Groups containing significant differences are marked with an asterisk ($p < 0.05$; $n = 3$; Tukey's test).

Wall Material	Encapsulation Method	Emulsifier	EE %	±SE	z	*p*
GA	FD	None	2.76	0.05	-	-
GA	FD	T20	1.40	0.42	-0.932	0.999
GA	FD	T80	1.10	0.14	-1.141	0.993
GA	SD	None	0.18	0.001	-	-
GA	SD	T20	26.99	0.85	18.483	<0.001*
GA	SD	T80	15.15	0.56	10.317	<0.001*
MD	FD	None	0.67	0.02	-	-
MD	FD	T20	2.64	0.26	1.354	0.972
MD	FD	T80	1.68	0.09	0.685	1.000
MD	SD	None	0.12	0.003	-	-
MD	SD	T20	2.43	0.15	1.493	0.912
MD	SD	T80	16.84	3.36	11.529	<0.001*

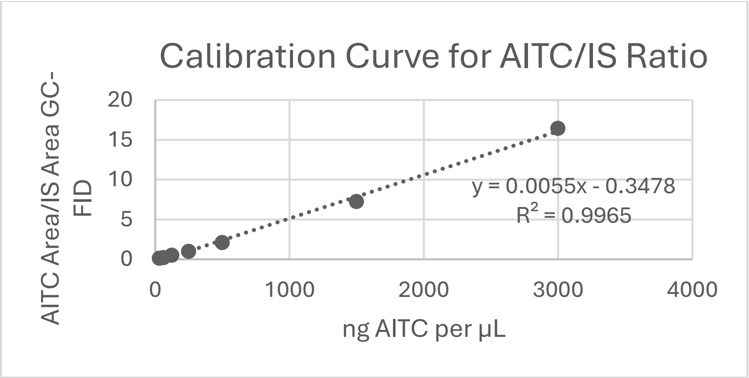

Figure S1 Calibration curve for allyl isothiocyanate (AITC) at concentrations ranging from 31.25 to 3000 ng/µL, spiked with 25 µL of 4960 ng/µL phenyl isothiocyanate (PITC) internal standard (IS). Data was acquired performing Gas Chromatography–Flame Ionization Detector (GC-FID) analysis and the curve was generated by plotting the peak area ratios of AITC to PITC against the standard AITC concentrations prepared via serial dilutions.

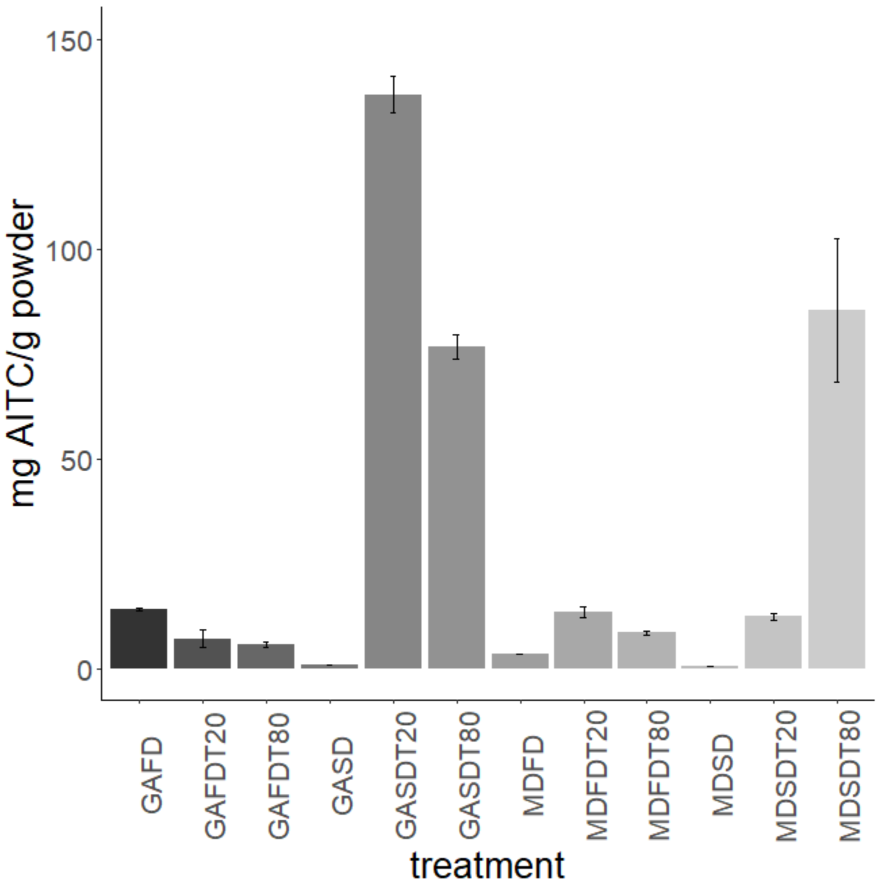

Figure S2. Summary of the mean (n = 3) encapsulation efficiency of allyl isothiocyanate (AITC) microcapsules (mg AITC/g powder) ± SE produced using freeze-drying (FD) and spray-drying (SD) with different formulations. Encapsulating agents include gum Arabic (GA) and maltodextrin (MD), with emulsifiers Tween-20 (T20) and Tween-80 (T80).

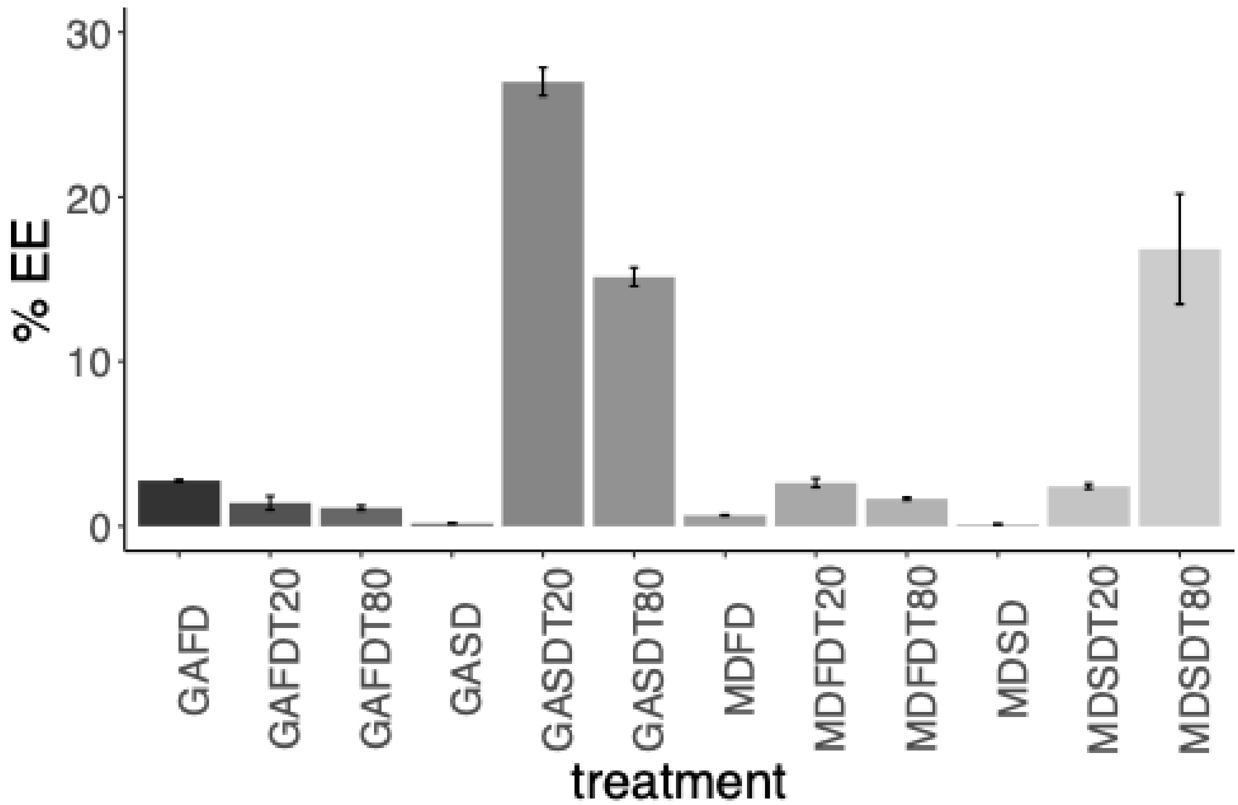

Figure S3. Summary of the mean (n = 3) encapsulation efficiency of allyl isothiocyanate (AITC) microcapsules (%) ± SE produced using freeze-drying (FD) and spray-drying (SD) with different formulations. Encapsulating agents include gum Arabic (GA) and maltodextrin (MD), with emulsifiers Tween-20 (T20) and Tween-80 (T80).

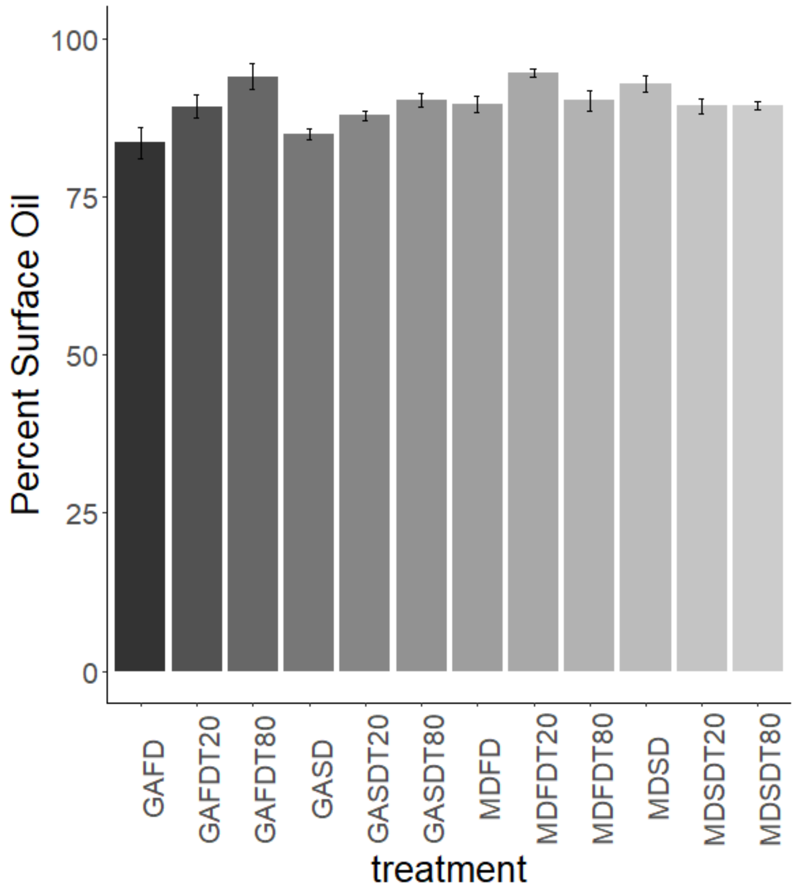

Figure S4. Summary of the mean (n = 3) percentage surface oil of allyl isothiocyanate (AITC) microcapsules (%) ± SE produced using freeze-drying (FD) and spray-drying (SD) with different formulations. Encapsulating agents include gum Arabic (GA) and maltodextrin (MD), with emulsifiers Tween-20 (T20) and Tween-80 (T80).
